# Hématome sous dural chronique bilatéral compliquant une dérivation ventriculo-péritonéale

**DOI:** 10.11604/pamj.2015.20.391.6671

**Published:** 2015-04-21

**Authors:** Rachid Ammor, Omar Boulahroud

**Affiliations:** 1 Neurochirurgie, Hopital militaire My Ismail, Meknes, Maroc

**Keywords:** hématome sous dural chronique, DVP, valve moyenne pression, chronic subdural hematoma, DVP, medium pressure valve

## Image en medicine

Un enfant de 12 ans, sans antécédents, a été hospitalisé dans notre formation pour des céphalées frontales évoluant depuis deux mois compliquées de vomissements depuis trois semaines. L'IRM cérébrale a objectivé une hydrocéphalie triventriculaire sur sténose non tumorale de l'aqueduc de Sylvius (A). Une valve ventriculo-péritonéale type moyenne pression fixe à été mise en place. L'évolution post opératoire a été favorable. Le contrôle clinique à 6 mois était sans particularité alors que le scanner de contrôle (B) a objectivé un hématome sous dural chronique hémisphérique bilatéral. Vu la tolérance clinique, une surveillance clinique et tomodensitométrique a été adoptée. Le scanner de contrôle réalisé deux mois plus tard a objectivé la quasi-disparition des deux collections (C). L'hématome sous dural chronique est une complication rare de la dérivation ventriculo-péritonéale. L'évacuation chirurgicale de la collection avec révision de la valve est la règle dans les formes symptomatiques. Les cas asymptomatiques doivent inciter à faire des contrôles cliniques et scannographiques rapprochés.

**Figure 1:**
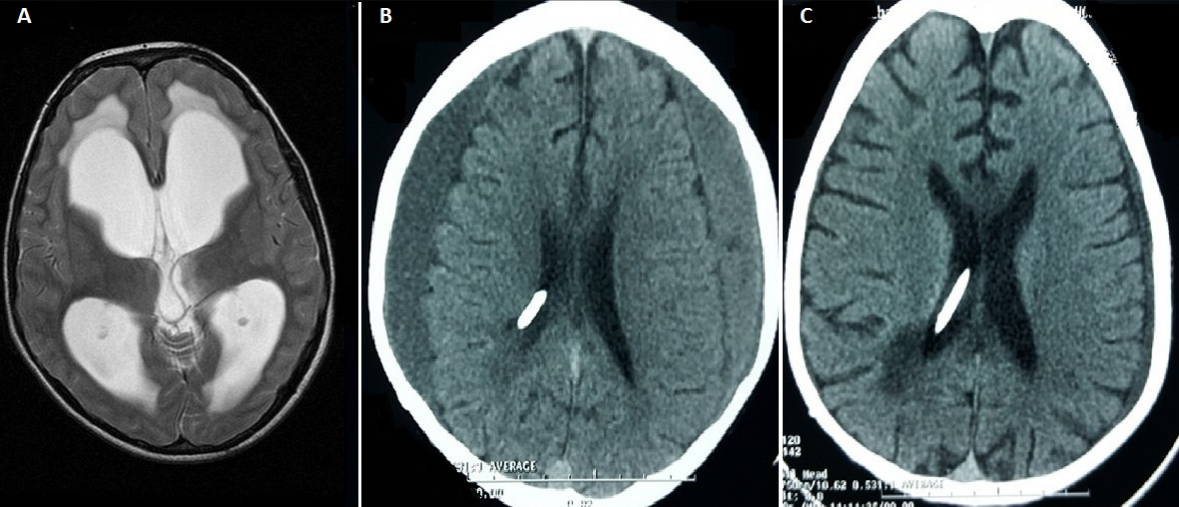
(A) coupe axiale en séquence pondérée T2 objectivant la dilatation triventriculaire; (B) contrôle TDM à 6 mois du postopératoire montrant la collection sous durale bilatérale; (C) contrôle TDM à 8 mois montrant la disparition de la collection de droite et la persistance d'une petite lame de l'hématome sous dural chronique à gauche

